# Acute obstructive fungus ball pyelonephritis with *Candida parapsilosis*: A case report

**DOI:** 10.1002/ccr3.7484

**Published:** 2023-06-09

**Authors:** Rajo Païdia Radinasoa, Armel Mamihaja Andrianiaina, Haingotiana Randriamamizoly, Luck Franscisca Adrien Andrianarivony, Harilalaina Willy Franck Randriamarotia, Hanta Marie Danielle Vololontiana

**Affiliations:** ^1^ Departement of Internal Medecine «Pavillon Spécial B» University Hospital of Antananarivo Antananarivo Madagascar; ^2^ Departement of Nephrology University Hospital of Antananarivo Antananarivo Madagascar

**Keywords:** *Candida parapsilosis*, fungus ball, pyelonephritis

## Abstract

**Key Clinical Message:**

Fungal lithiasis is a rare but serious complication of candiduria. Frequent use of broad‐spectrum antibiotics is a contributing factor in predisposed subjects. Two CBEUs are required to confirm the diagnosis of candiduria. Anti‐fungal is found effective to eradicate the fungus ball besides surgery.

**Abstract:**

Lithiasis by fungus ball is a serious complication of candiduria. Our case was a 58‐year‐old man who presented an acute obstructive pyelonephritis. Ultrasound revealed a left ureteral lithiasis. Biological examination revealed *Candida parapsilosis*. Antifungal was effective with good evolution. Broad‐spectrum antibiotic therapy is one favoring factor.

## 1 INTRODUCTION

Candida urinary tract infection is actually on the rise, especially in hospitals. The most frequent species found among candidiasis is *Candida albicans* and rarely *Candida parapsilosis*. The emergence of new species of Candida with an increase in their resistance to antifungal drugs is the reason for this.^1^ A simple urinary colonization is difficult to differentiate from a proper urinary infection. Factors have been found to favor infection, such as immunodepression, diabetes, prostheses, or endogenous surgical material, taking broad‐spectrum antibiotic therapy.^2^ It is a serious infection because it can range from a simple asymptomatic candiduria to a troublesome cystitis or even an obstructive pyelonephritis by the formation of candidal granuloma or “fungus ball” which is the most serious and can engage the vital prognosis of the patient in the short term.^3^ Our objective is to report a case of obstructive pyelonephritis caused by a *Candida parapsilosis* fungus ball and to discuss the factors that may contribute to it.

## 2 CASE PRESENTATION

**FIGURE 1:**
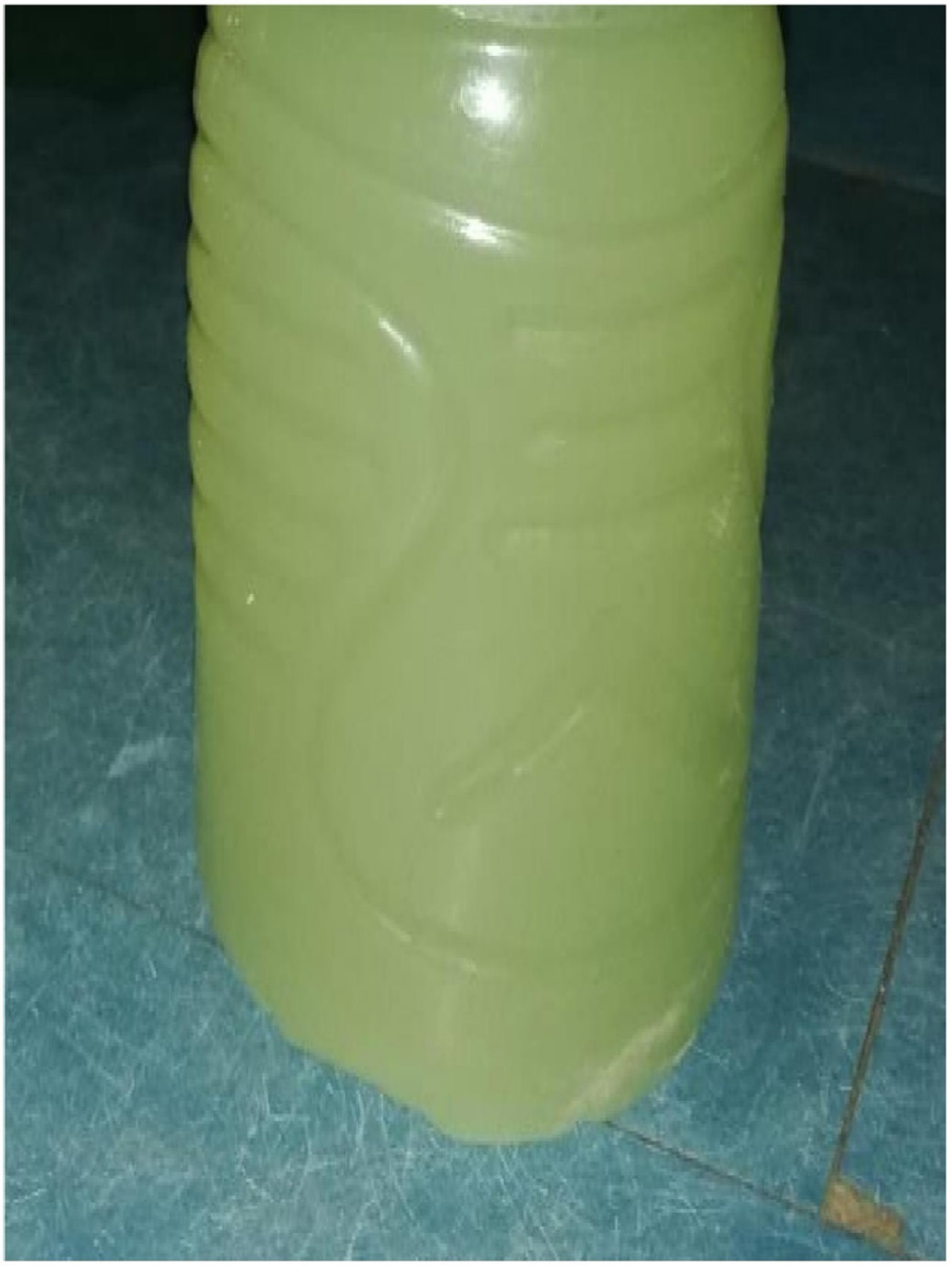
Urinary macroscopic appearance of candiduria « cloudy urine with whitish precipitation ».

**FIGURE 2:**
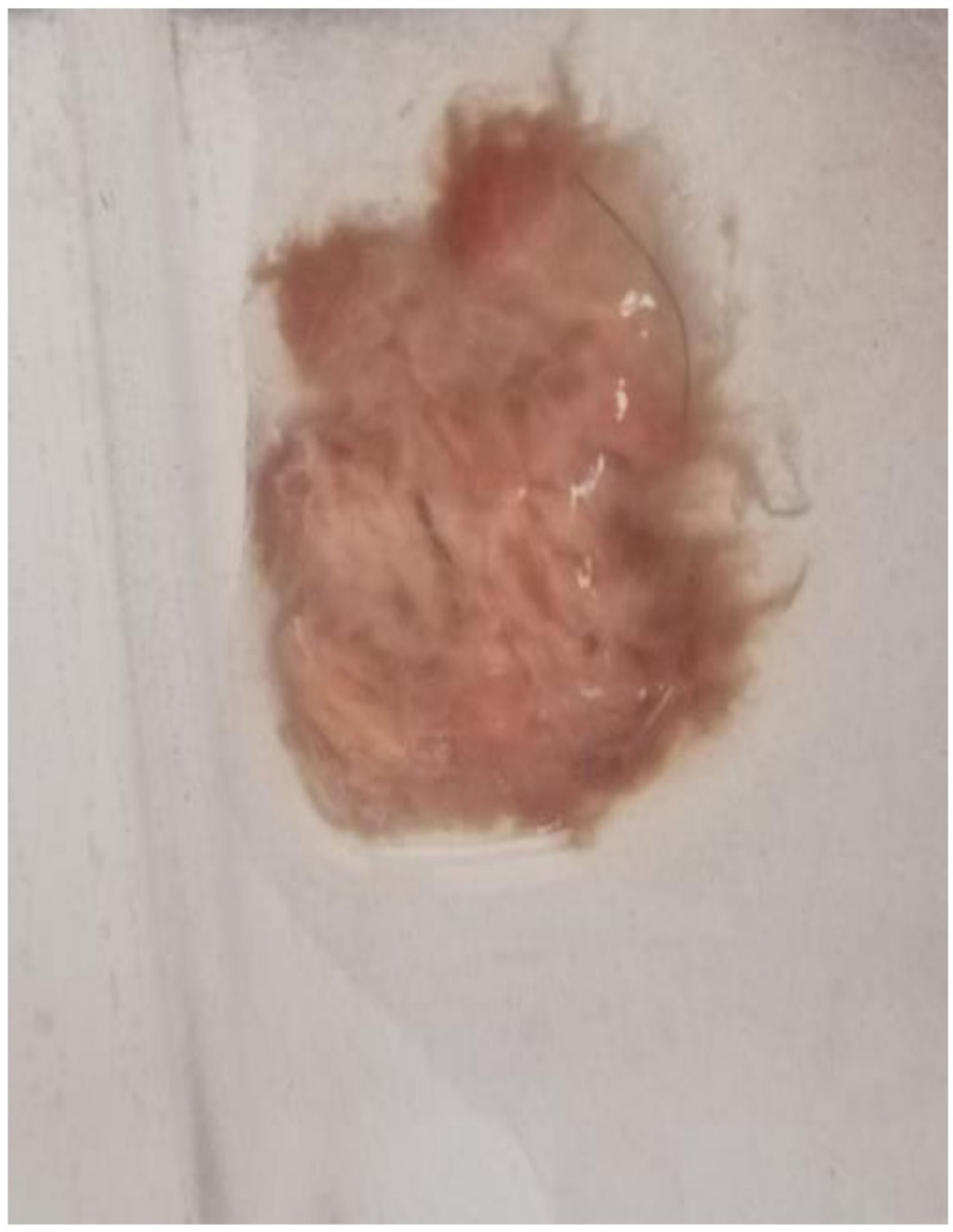
Macroscopic view of a fungus ball « squidgy and hairy appearance lithiasis ».

**FIGURE 3:**
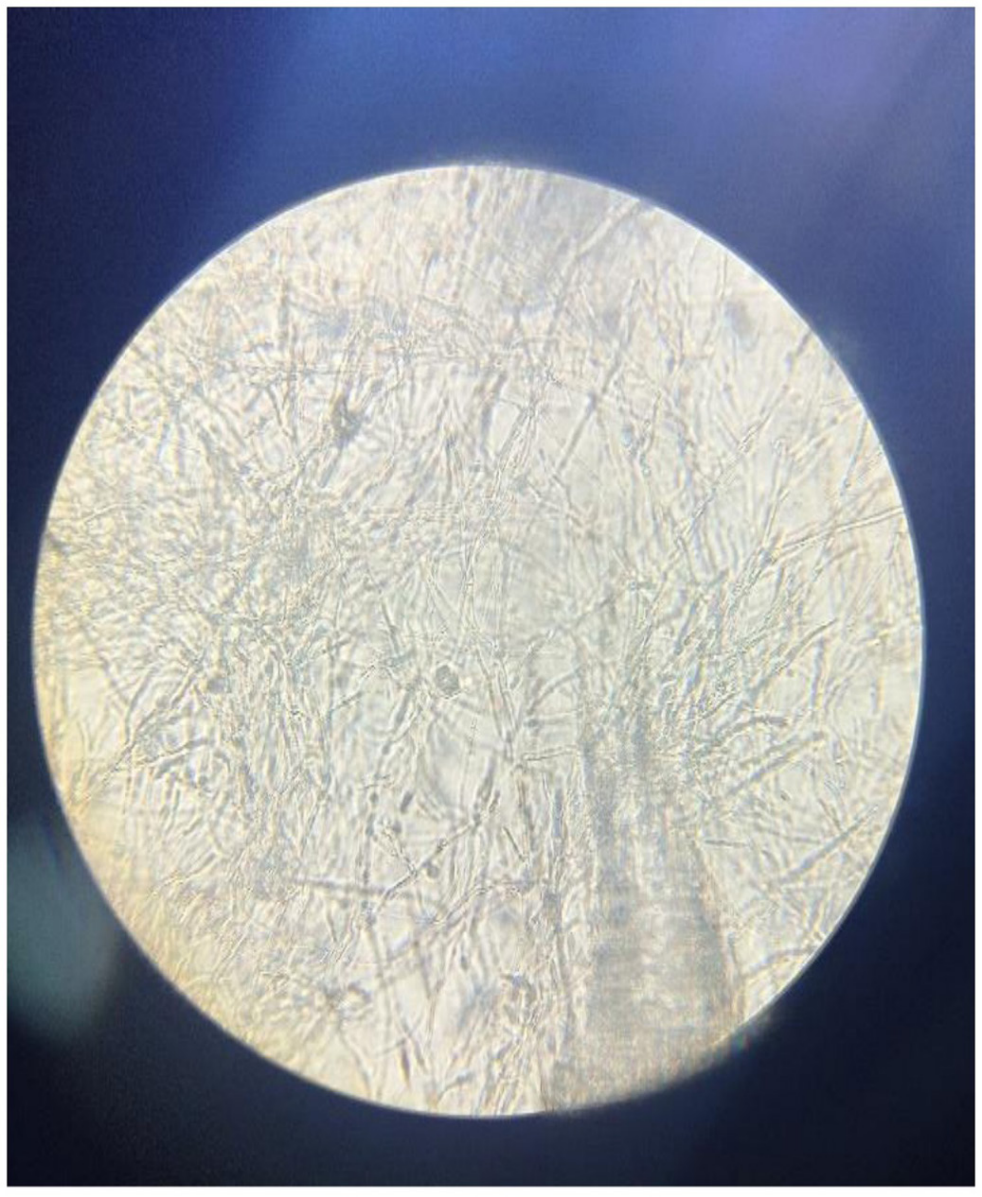
*Candida parapsilosis* seen under the light microscope « non‐smooth colonies with pseudohypal cells ».

A 58‐year‐old man was admitted to the nephrology department for mictional burning and pollakiuria evolving for 2 weeks. The patient is a poorly treated gouty and hypertensive, not diabetic, and occasionally ethylic. He has a history of recurrent urinary tract infection and is frequently treated with antibiotics for 1 year. The last cytobacteriological examination of the urine (CBEU) showed a Quinolone‐resistant *Escherichia coli* infection 1 month before his admission. About surgical history: he has a double J catheterization in October 2020 with the removal of a left calcium lithiasis and a left renal cystectomy, removal of the double J catheter in 2021. During his hospitalization, the patient presented pollakiuria and mictional burning with a preserved diuresis of 2 L/24 h with clear urine, all evolving in an apyretic context. The biological examinations found a biological inflammatory syndrome with a neutrophilic hyperleukocytosis (Table 1), an acute renal failure stage 2 of AKIN, an elevated uric acid level at 511 μmoL/L, uremia at 22 mmoL/L, without hypocalcemia or ionic disorder. After, he presented a left renal colic with a positive Giordano sign and cloudy urine with whitish precipitation (Figure 1). The ultrasound showed a left ureteral lithiasis image of 13.5*7.4 mm with left pyelocalic dilatation and thickening of the bladder wall. CBEU revealed non‐albicans candida candiduria in the first sample (Table 2). A second sample was taken 2 days later. A worsening of the condition was marked by an obstructive acute kidney failure with creatinine at 950 μmoL/L and uremia at 53 mmoL/L with hyperkalemia at 7.5 mmoL/L. We initiated drug management of the hyperkalemia with monitoring of renal function and ionogram. The patient had a urinary debacle of 3.5 L with hematuria and expulsion of non‐calcium debris (Figure 2), which was analyzed in the laboratory, followed by the disappearance of the left renal colic and negative Giordano sign. The follow‐up ultrasound showed no more left urinary lithiasis but the persistence of the thickening of the bladder wall with a blood clot at the bladder fundus. The renal function had improved within 24 h with a decrease in creatinine at 720 μmoL/L, urea at 44 mmoL/L, and a normalization of the ionogram (Table 1). The second cytobacteriological and mycosal examination of the urine confirmed the presence of *Candida parapsilosis* candiduria sensitive to: Amphotericin B, fluconazole, and ketoconazole. The direct examination between the slide and slab of the specimen from the urine debacle also confirmed the nature of a *Candida parapsilosis* fungus ball (Figure 3). We thus retained the diagnosis of a lithiasis pyelonephritis on a candida parapsilosis fungus ball. As a treatment, we started a fluconazole‐type antifungal agent at a dose of 200 mg per day on day 1 then 100 mg per day according to the adaptation with the renal function for a duration of 3 weeks; adenuric 40 mg per day for the gout. The evolution of the patient under treatment was marked by the disappearance of mictional burning and pollakiuria, a normal diuresis, and a return to normal renal function. An improvement of the biological inflammatory syndrome after 14 days of treatment was marked with a negativation of the cytobacteriological and mycosal examination of urine.

**TABLE 1:** Biological data during the patient's hospitalization.

	06‐05‐2022	13/05/2022	18/05/2022	20/05/2022
Leukocytes	12.20 G/L	16.7 G/L	19.8 G/L	17 G/L
Neutrophils	8.5 G/L	14.2 G/L	17.2 G/L	15 G/L
Urea	22.5 mmol/L	34.90 mmol/L	53 mmol/L	50 mmol/L
Creatinine	430 μmol/L	387 μmol/L	917 μmol/L	724 μmol/L
CRP	68 mg/L	119.6 mg/L	150 mg/L	130 mg/L

**TABLE 2:** Urine cytobacteriological test results.

	21/04/2022	06/05/2022	13/05/2022	30/05/2022
Appearance	Citrine Yellow	Cloudy	Cloudy	Citrine Yellow
Leukocytes	20,000/ml	50,000/mL	11,450,900/mL	35,000/mL
Red blood cells	<1000	2000/mL	3400 mL	<1000
Epithelial cells	Absent	Absent	Absent	Absent
Urinary cylinders	Absent	Absent	Absent	Absent
Crystals	Absent	Absent	Absent	Absent
Germs	Esherichia coli	Candida no albicans	Candida parapsilosis	Absent

## 3 DISCUSSION

Candida urinary tract infection is difficult to differentiate from simple urinary colonization. It is reported in the literature that the most frequent manifestation of candiduria is pyelonephritis followed by signs of cystitis and others.^4^ The examination key to confirm the diagnosis of candiduria is the cytobacteriological examination of the urine to be done twice, with significant leucocyturia, the presence of candida yeast with identification of the species, and a positive culture on Sabouraud medium.^5^ Ultrasound and CT scan of the urinary tract are useful to look for possible lithiasis (fungal ball) but the confirmation is the anatomopathological and mycological examination.^6^ *Candida parapsilosis* is responsible for 4.3% of candidiasis,^7^ this specie is commensal of the skin, especially the hands, and multiplies easily on plastic materials at the temperature of 20 to 40°C.^8^ Several factors have been described as favoring candida urinary tract infections such as immunosuppression (HIV, chronic renal failure, cancer, diabetes, and the presence of intrabody prosthetic material). The use of long‐term corticosteroids and immunosuppressive drugs was also reported in the literature.^9^ The frequent use of broad‐spectrum antibiotics is one of the main causes found.^10^ A fungus ball can originate from agglutination of a necrotic nucleus tissue (papillary necrosis), mucosus debris, and foreign or lithiasic debris; this can then lead to a urinary tract obstruction and hydronephrosis, which is a very rare complication of candiduria. The most frequently observed pathogens are *C. albicans* and *C. tropicalis*, but *Aspergillus flavus*, *Aspergillus nidulans*, *Rhizopus*, and *C. parapsilosis* also are reported.^11^ Our case presented with a clinical board of cystitis complicated by lithiasis pyelonephritis on ultrasound with a first positive CBEU for non‐albicans candida. The urinary debacle had expelled the obstacle which was in fact a fungal ball confirmed by mycological examination with immediate ultrasound control without lithiasis found. The second CBEU confirmed the identity of the strain of *Candida parapsilosis* with an antifungiogram without resistance to the usual antifungals. The responsible factors found in our case were frequent use of broad‐spectrum antibiotics, previous placement of a DJ stent, and possible underlying chronic renal failure. The differential diagnoses in front of the fungus ball are calcic lithiasis and uric acid lithiasis. We need to look for a candida infection in subjects who has a severe urinary tract infection with a high‐risk factor. Do not use broad‐spectrum antibiotics without clear bacteriologic evidence in subjects who has risk factors for fungal infection. For patients with symptomatic candida urinary tract infections, a variety of treatment options are available. Fluconazole is the antifungal agent of choice, achieving high urine concentrations with the oral formulation at the posology 200–400 mg per day for 14 days.^12^ Treatment with percutaneous lithotomy has been found especially in children.^13^

A possible systematic treatment with antifungal agents in subjects at risk would be an efficient way to decrease the recrudescence of fungal infections in hospitals.^14^

## 4 CONCLUSION

Candida urinary tract infection is one of the candidiasis currently on the rise in hospitals. It occurs in patients with already defined immunodepressive factors, either because the patient is deficient or because of iatrogenic causes. The clinical forms are often pyelonephritis and cystitis. The occurrence of obstruction by fungal lithiasis should not be overlooked. Two cytobacteriological and mycosal examination of urine are necessary to make the diagnosis, with imaging if necessary. The treatment is based on the identified species and the antifungal.

## AUTHOR CONTRIBUTIONS

**Rajo Païdia Radinasoa:** Conceptualization; data curation; investigation; project administration; writing – original draft. **Armel Mamihaja Andrianiaina:** Funding acquisition; investigation; methodology; resources. **Haingotiana Randriamamizoly:** Data curation; formal analysis; investigation; methodology; writing – review and editing. **Adrien Andrianarivony:** Resources; software; visualization; writing – review and editing. **Harilalaina Willy Franck Randriamarotia:** Supervision; validation; visualization; writing – review and editing. **Hanta Marie Danielle Vololontiana:** Supervision; validation; visualization.

## CONFLICT OF INTEREST STATEMENT

We do not have any conflict of interest.

## CONSENT STATEMENT

Written informed consent was obtained from the patient to publish this report in accordance with the journal's patient consent policy.

## Data Availability

Data available within the article or its supplementary materials. The authors confirm that the data supporting the findings of this study are available within the article and/or its supplementary materials.
